# Impact of istradefylline on motor and non-motor symptoms in patients with Parkinson’s disease: Subanalysis of the ISTRA ADJUST PD

**DOI:** 10.1016/j.prdoa.2025.100327

**Published:** 2025-04-17

**Authors:** Hiroshi Nagayama, Osamu Kano, Renpei Sengoku, Naotake Yanagisawa, Asako Yoritaka, Keisuke Suzuki, Noriko Nishikawa, Yohei Mukai, Kyoichi Nomura, Norihito Yoshida, Morinobu Seki, Miho Kawabe Matsukawa, Hiroo Terashi, Katsuo Kimura, Jun Tashiro, Shigeki Hirano, Hidetomo Murakami, Hideto Joki, Tsuyoshi Uchiyama, Hideki Shimura, Kotaro Ogaki, Jiro Fukae, Yoshio Tsuboi, Kazushi Takahashi, Toshimasa Yamamoto, Kenichi Kaida, Ryoko Ihara, Kazutomi Kanemaru, Taku Hatano

**Affiliations:** a Department of Neurofunctional Science, Nippon Medical School, Tokyo, Japan; b Department of Neurology, Toho University Faculty of Medicine, Tokyo, Japan; c Department of Neurology, Daisan Hospital, The Jikei University School of Medicine, Tokyo, Japan; d Medical Technology Innovation Center, Juntendo University, Tokyo, Japan; e Department of Neurology, Juntendo University Koshigaya Hospital, Saitama, Japan; f Department of Neurology, Dokkyo Medical University Hospital, Tochigi, Japan; g Department of Neurology, Faculty of Medicine, Juntendo University, Tokyo, Japan; h Department of Neurology, National Center of Neurology and Psychiatry, Tokyo, Japan; i Department of Neurology, Higashimatsuyama Municipal Hospital, Saitama, Japan; j Department of Neurology, Saitama Medical Center, Saitama Medical University, Saitama, Japan; k Department of Neurology, Keio University School of Medicine, Tokyo, Japan; l Department of Neurology, Tokyo Metropolitan Institute for Geriatrics and Gerontology, Tokyo, Japan; m Department of Neurology, Tokyo Medical University, Tokyo, Japan; n Department of Neurology, Yokohama City University Medical Center, Yokohama, Japan; o Sapporo Parkinson MS Neurological Clinic, Sapporo, Japan; p Department of Neurology, Graduate School of Medicine, Chiba University, Chiba, Japan; q Department of Neurology, The Jikei University School of Medicine, Tokyo, Japan; r Department of Neurology, National Hospital Organization Yokohama Medical Center, Yokohama, Japan; s Department of Neurology, Seirei Hamamatsu General Hospital, Hamamatsu, Japan; t Department of Neurology, Juntendo University Urayasu Hospital, Chiba, Japan; u Department of Neurology, Juntendo University Nerima Hospital, Tokyo, Japan; v Department of Neurology, Fukuoka University, Fukuoka, Japan; w Department of Neurology, Tokyo Metropolitan Neurological Hospital, Tokyo, Japan; x Department of Neurology, Saitama Medical University, Saitama, Japan

**Keywords:** Adenosine A_2A_ receptor antagonist, Istradefylline, Levodopa, Motor symptoms, Non-motor symptoms, Parkinson’s disease

## Abstract

**Introduction:**

Parkinson’s disease (PD) presents with diverse motor and non-motor symptoms, some of which do not fully respond to dopamine replacement therapy. To clarify the effects of the adenosine A_2A_ receptor antagonist istradefylline (IST) on PD symptoms, we conducted a subanalysis of the 37-week ISTRA ADJUST PD randomized controlled trial.

**Methods:**

Patients with PD experiencing wearing-off with levodopa at 300–400 mg/day were randomized 1:1 to receive IST or no IST (control). Levodopa doses were titrated according to clinical severity throughout the study. PD symptoms were assessed using the Movement Disorder Society Unified Parkinson’s Disease Rating Scale (MDS-UPDRS) and Parkinson’s Disease Questionnaire-39 (PDQ-39).

**Results:**

The efficacy analyses included 105 patients (IST, n = 52; control, n = 53). Regarding MDS-UPDRS subitems in patients in the IST group, significant improvements (*p* < 0.05) were observed at week 36 compared with baseline for both motor (e.g., facial expression, rigidity, finger tapping, toe tapping, time spent in the off state, and complexity of motor fluctuation) and non-motor (e.g., daytime sleepiness, fatigue, and doing hobbies and other activities) symptoms. In patients in the control group, significant improvements (*p* < 0.05) were observed for motor symptoms only. Regarding PDQ-39 subitems in patients in the IST group, significant improvements (*p* < 0.05) were observed at week 36 compared with baseline in doing up buttons or shoelaces, writing, feeling angry or bitter, and unexpectedly falling asleep during the day.

**Conclusion:**

IST can improve motor and non-motor symptoms and quality of life suggesting that it provides more comprehensive improvement in PD symptoms than increasing the levodopa dose alone.

**Clinical trial registration:**

Japan Registry of Clinical Trials; study ID: jRCTs031180248.

## Highlights

• In Parkinson’s patients, off episode symptoms do not fully improve with levodopa.
• Istradefylline is an adjunct to levodopa for patients experiencing off episodes.
• Istradefylline improved motor and non-motor symptoms in a controlled clinical trial.
• This adjunct treatment also enhances quality of life measures for patients.
• Istradefylline improved symptoms better than increasing the levodopa dose alone.

## 1 Introduction

Parkinson’s disease (PD) is a neurodegenerative disorder characterized by motor dysfunction associated with degenerative loss of dopamine-producing neurons in the substantia nigra. However, PD symptoms are now recognized as heterogeneous and accompanied by clinically meaningful non-motor symptoms. The characteristic motor symptoms are resting tremors, bradykinesia, rigidity, and poor postural reflexes, whereas common non-motor symptoms are cognitive impairment, mood disorders, sleep disturbances, hallucinations, autonomic dysfunction (such as constipation and orthostatic hypotension), and sensory disturbances (such as visual problems and pain) [1]. This indicates that the pathology of PD affects numerous areas of the nervous system and involves a variety of neurotransmitters and protein aggregates beyond just Lewy bodies [2]. Non-motor symptoms such as depression are also fundamentally closely related to the pathology of PD [3], increasing the complexity of managing PD and impacting patients’ quality of life (QOL). Notably, non-motor symptoms frequently appear before motor impairments.

The main treatment for PD is dopamine replacement therapy, with levodopa being the most effective option for managing motor symptoms. However, prolonged use of levodopa leads to motor complications, including wearing-off and dyskinesia [4]. Moreover, dopamine replacement therapy is ineffective for some motor and non-motor symptoms. Therefore, in addition to levodopa, adjunctive therapies using other anti-parkinsonian medications with non-dopaminergic mechanisms are valuable for the treatment of PD [5]. As one of these important therapeutic options, the adenosine A_2A_ receptor antagonist istradefylline (IST) is used in combination with levodopa to improve wearing-off/OFF episodes in patients with PD [6]. IST inhibits the binding of adenosine to adenosine A_2A_ receptors in the striatum and globus pallidus, suppressing the excitation of GABA neurons. This action helps restore balance in the basal ganglia-thalamocortical circuit, offering a non-dopaminergic approach to relieving motor symptoms in patients with PD [7]. In addition, based on its mechanism of action and the findings from non-clinical studies and short-term trials involving small groups of patients, IST is also expected to improve non-motor symptoms such as sleep disturbances and depression [[8], [9], [10], [11], [12], [13], [14], [15]]. However, the expected efficacy for non-motor symptoms and QOL remains to be confirmed. Therefore, it is clinically important to examine the effect of IST on non-motor symptoms and QOL, in particular, and not just motor symptoms.

The results of the ISTRA ADJUST PD study [5] showed that IST significantly improved the scores of Parts I, III, and IV of the Movement Disorder Society Unified Parkinson’s Disease Rating Scale (MDS-UPDRS) [16] as well as device-evaluated motor activities. These findings suggest that IST may improve not only motor symptoms but also non-motor symptoms in patients with PD experiencing wearing-off. In the present study, we performed a detailed evaluation of the subitems of both the MDS-UPDRS and the Parkinson’s Disease Questionnaire-39 (PDQ-39) as a subanalysis of the ISTRA ADJUST PD study [5]. Our goal was to gain a more comprehensive understanding of the impact of IST on non-motor symptoms and QOL as well as motor symptoms in patients with PD. In this paper, we report the effects of IST with a particular focus on non-motor symptoms.

## 2 Materials and methods

The specifics regarding the methods are available in previously published papers [5,17].

### 2.1 Study design

This 37-week, multicenter, open-label, randomized, parallel-group-controlled study (the ISTRA ADJUST PD study) was performed at 20 sites in Japan from February 2019 to May 2022 (registration period: May 2019–November 2020). The study protocol (Version 6.0, 1 November 2021) and all other study documentation (approval number CRB3180012) were reviewed and approved by the Juntendo University Certified Review Board (J18-006). This study was registered with the Japan Registry of Clinical Trials (jRCTs031180248). All patients provided written informed consent prior to study enrollment.

### 2.2 Patient participants

Eligible patients were aged 30 to 84 years, were taking levodopa three or more times daily (300–400 mg total), were experiencing wearing-off episodes, had been diagnosed with PD in accordance with the International Parkinson and Movement Disorder Society criteria, were at Hoehn and Yahr stage ≤ 3 (on), and had provided written consent. Patients taking other anti-PD drugs were also included. The exclusion criteria were prior IST treatment, study drug use within the past 4 months, dementia (score of ≤ 23 on the Japanese version of the Mini-Mental State Examination), prior PD neurosurgery, current/planned use of levodopa/carbidopa suspension use, recent PD treatment changes (within 4 weeks), use of strong CYP3A4 inhibitors within 14 days, moderate/severe hepatic disorder, pregnancy or lactation, or investigator’s judgment of ineligibility.

### 2.3 Treatments

The patients were randomly allocated to the IST group or control group (no IST treatment) at a 1:1 ratio using a central computer-based minimization method. Both the participants and study investigators were aware of the treatment assignments. The patients in the IST group began treatment at 20 mg daily, increasing to 40 mg at week 1 if tolerated and implementing dose reductions if needed. Additionally, the levodopa dose was increased by 50 mg/day at week 4 and every 4 weeks thereafter if the Clinical Global Impression-Severity scale (CGI-S) score was ≥ 4 on the day of observation. The patients in the control group had their levodopa dosage increased by 50 mg/day at week 0, with a potential increase of 50 mg/day every 4 weeks if the CGI-S score was ≥ 4 on the day of observation. Dose reductions for intolerable adverse reactions were allowed. The dose or regimen of other anti-PD drugs remained unchanged within 4 weeks prior to enrollment and throughout the 37-week treatment period, except for reductions due to intolerable adverse reactions.

### 2.4 Outcomes

The scores for the subitems of MDS-UPDRS Part I (non-motor experiences of daily living), Part II (motor experiences of daily living), Part III (motor examination), and Part IV (motor complications) as well as the PDQ-39 were evaluated at weeks 0, 12, 24, and 36 after treatment.

### 2.5 Statistical analysis

The efficacy population was used for all statistical analyses. As previously described [5,17], the sample size was set at 111 patients to ensure that 50 patients were evaluated in both groups in the efficacy population.

The Mann-Whitney *U* test was used to compare subitem scores at weeks 0 and 36 between groups. Changes in subitem scores from baseline (week 0) were evaluated using the Wilcoxon signed-rank test. A two-sided 5 % significance level was set for between-group and between-timepoint comparisons, and two-sided 95 % confidence intervals were calculated. No imputation was made for missing data. We prepared a detailed statistical analysis plan before the database was finalized and locked. SAS version 9.4 (SAS Institute Inc., Cary, NC, USA) was used for the statistical analyses.

## 3 Results

### 3.1 Patients

The patients’ details have been previously described [5]. Between May 2019 and November 2020, 115 patients were recruited. Of these, one patient declined to participate prior to allocation, resulting in 114 patients being randomized to the IST or control group (57 patients per group). After enrollment and allocation, five patients in the IST group were withdrawn from the study because of not starting treatment (n = 5), withdrawal of consent (n = 4), or withdrawal by the study investigator (n = 5), four patients in the control group were withdrawn because of not meeting the inclusion criteria (n = 2), withdrawal of consent (n = 3), or withdrawal by the study investigator (n = 4). Note that some patients experienced multiple reasons for withdrawal. Therefore, the efficacy population comprised 52 and 53 patients in the IST group and control group, respectively [5]. No notable imbalances in the baseline demographic and clinical characteristics were observed between the two groups (Table 1).

**Table 1:** Patient characteristics.

	IST group (n = 52)	Control group (n = 53)
Age (years)	65.6 ± 8.9	65.0 ± 8.6
Sex, male	26 (50.0)	23 (43.4)
Duration of PD (years)	6.3 ± 3.5	6.8 ± 3.4
Age at onset of PD (years)	59.5 ± 9.3	58.5 ± 9.1
Duration of wearing-off phenomena (years)	1.2 ± 1.4	1.5 ± 1.7
Dyskinesia	12 (23.1)	10 (18.9)
Duration of levodopa therapy (years)	4.1 ± 2.7	4.2 ± 2.6
Levodopa dose (mg/day)	336.5 ± 45.5	337.7 ± 45.9
Levodopa equivalent dose (mg/day)	533.5 ± 179.2	546.5 ± 173.8
mH&Y (on)		
Stage 0	1 (1.9)	2 (3.8)
Stage 1	2 (3.8)	4 (7.5)
Stage 1.5	3 (5.8)	1 (1.9)
Stage 2	27 (51.9)	29 (54.7)
Stage 2.5	9 (17.3)	11 (20.8)
Stage 3	10 (19.2)	6 (11.3)
Stage 4	0 (0.0)	0 (0.0)
Stage 5	0 (0.0)	0 (0.0)
mH&Y (off)		
Stage 0	0 (0.0)	0 (0.0)
Stage 1	0 (0.0)	2 (3.8)
Stage 1.5	1 (1.9)	1 (1.9)
Stage 2	11 (21.2)	14 (26.4)
Stage 2.5	12 (23.1)	13 (24.5)
Stage 3	22 (42.3)	21 (39.6)
Stage 4	5 (9.6)	2 (3.8)
Stage 5	1 (1.9)	0 (0.0)
MDS-UPDRS Part I	9.4 ± 4.4	8.6 ± 4.2
MDS-UPDRS Part II	11.6 ± 6.2	10.7 ± 6.0
MDS-UPDRS Part III	21.6 ± 12.8	20.6 ± 13.1
MDS-UPDRS Part IV	4.9 ± 2.3	4.7 ± 1.9
PDQ-39	26.3 ± 20.5	22.9 ± 18.9
Concomitant anti-PD drugs		
Dopamine agonist	29 (55.8)	38 (71.7)
MAOB inhibitor	29 (55.8)	25 (47.2)
COMT inhibitor	16 (30.8)	11 (20.8)
Zonisamide	12 (23.1)	17 (32.1)
Amantadine	2 (3.8)	7 (13.2)
Anticholinergic agents	4 (7.7)	6 (11.3)
Droxidopa	0 (0.0)	1 (1.9)

This table was adapted from a previous paper [5].

Data are presented as n (%) or mean ± SD.

There were no significant differences (*p* ≥ 0.05) between the two groups for any of the items.

COMT, catechol-O-methyltransferase; IST, istradefylline; MAOB, monoamine oxidase-B; MDS-UPDRS, Movement Disorder Society Unified Parkinson’s Disease Rating Scale; mH&Y, modified Hoehn and Yahr; PD, Parkinson’s disease; PDQ, Parkinson’s Disease Questionnaire; SD, standard deviation

### 3.2 MDS-UPDRS

No significant differences were found in each score at weeks 0 and 36 between the IST and control groups [5].

The total scores of the MDS-UPDRS Part I, III, and IV were significantly improved in the IST group (*p* < 0.05), whereas those of the MDS-UPDRS Part III and IV were significantly improved in the control group at week 36 compared with baseline (*p* < 0.05) [5] (Table 2, Supplementary Tables S1−S4).

**Table 2:** Change in MDS-UPDRS and PDQ-39 scores from weeks 0 to 36.

Endpoint		Change from weeks 0 to 36 median (min, Q1, Q3, max)	
		IST group	Control group
MDS-UPDRS Part I	Total	−1.0 (−9, −3.0, 0.0, 6)^a^	0.0 (−8, −1.0, 2.0, 10)
	Apathy	0.0 (−2, 0.0, 0.0, 0)	0.0 (0, 0.0, 0.0, 2)^a^
	Daytime sleepiness	0.0 (−2, −1.0, 0.0, 2)^a^	0.0 (−2, 0.0, 0.0, 3)
	Fatigue	0.0 (−2, −1.0, 0.0, 1)^a^	0.0 (−1, 0.0, 0.5, 3)
MDS-UPDRS Part II	Total	0.0 (−12, −3.0, 2.0, 8)	0.0 (−10, −3.0, 2.0, 7)
	Engaging in hobbies and other activities	0.0 (−4, −1.0, 0.0, 1)^a^	0.0 (−3, 0.0, 0.0, 1)
MDS-UPDRS Part III	Total	−4.0 (−22, −8.0, −1.0, 12)^a^	−1.5 (−17, −5.5, 2.0, 11)^a^
	Facial expression	0.0 (−2, −1.0, 0.0, 1)^a^	0.0 (−2, 0.0, 0.0, 1)
	Rigidity (right upper limb)	0.0 (−2, −1.0, 0.0, 1)^a^	0.0 (−2, 0.0, 0.0, 2)
	Rigidity (left lower leg)	0.0 (−2, −1.0, 0.0, 2)	0.0 (−2, −1.0, 0.0, 2)^a^
	Finger tapping (right)	0.0 (−2, −1.0, 0.0, 1)^a^	0.0 (−2, 0.0, 0.0, 1)
	Toe tapping (right)	0.0 (−2, −1.0, 0.0, 1)^a^	0.0 (−2, −0.5, 0.0, 2)
	Toe tapping (left)	0.0 (−3, −1.0, 0.0, 1)^a^	0.0 (−1, 0.0, 0.0, 1)
MDS-UPDRS Part IV	Total	−1.0 (−8, −2.0, 1.0, 7)^a^	−1.0 (−6, −3.0, 0.0, 4)^a^
	Time spent with dyskinesias	0.0 (−1, 0.0, 0.0, 3)^a^	0.0 (−2, 0.0, 0.0, 3)
	Time spent in the off state	−0.5 (−3, −1.0, 0.0, 1)^a^	0.0 (−2, −1.0, 0.0, 1)^a^
	Functional impact of fluctuations	0.0 (−4, −1.0, 1.0, 2)	0.0 (−2, −1.0, 0.0, 1)^a^
	Complexity of motor fluctuations	0.0 (−3,–1.0, 0.0, 1)^a^	0.0 (−4, −1.0, 0.0, 3)
PDQ-39	Total	0.0 (−64, −11.0, 6.0, 35)	0.0 (−28, −6.0, 4.0, 44)
Due to having Parkinson’s disease, how often during the last month have you…			
	Had problems doing up your buttons or shoelaces?	0.0 (−3, −1.0, 0.0, 1)^a^	0.0 (−1, 0.0, 0.0, 2)
	Had problems writing clearly?	0.0 (−3, −1.0, 0.0, 1)^a^	0.0 (−1, 0.0, 0.0, 2)
	Felt angry or bitter?	0.0 (−3, 0.0, 0.0, 1)^a^	0.0 (−1, 0.0, 0.0, 2)
	Unexpectedly fallen asleep during the day?	0.0 (−3, −1.0, 0.0, 1)^a^	0.0 (−3, −1.0, 0.0, 1)

IST, istradefylline; max, maximum; MDS-UPDRS, Movement Disorder Society Unified Parkinson’s Disease Rating Scale; min, minimum; PDQ, Parkinson’s Disease Questionnaire; Q, quartile.

a *p* < 0.05, Wilcoxon signed-rank test (vs. baseline [week 0]).

The MDS-UPDRS Part I subitem scores at week 36 were compared with those at baseline (week 0) (Table 2, Supplementary Table S1). Daytime sleepiness and fatigue were significantly improved in the IST group (*p* < 0.05), whereas apathy was significantly worsened in the control group (*p* < 0.05). Table 2 and Supplementary Table S2 show the change in the MDS-UPDRS Part II subitem scores from weeks 0 to 36. Doing hobbies and other activities was significantly improved in the IST group (*p* < 0.05), whereas no significant improvement was observed in the control group. Among the MDS-UPDRS Part III subitem scores at week 36 compared with those at baseline, facial expression, rigidity (right upper limb), finger tapping (right), and toe tapping (right and left) were significantly improved in the IST group (*p* < 0.05), whereas rigidity (left lower leg) was significantly improved in the control group (*p* < 0.05) (Table 2, Supplementary Table S3). Table 2 and Supplementary Table S4 show the changes in MDS-UPDRS Part IV subitem scores from weeks 0 to 36. Time spent in the off state and complexity of motor fluctuations were significantly improved in the IST group (*p* < 0.05), whereas time spent in the off state and functional impact of fluctuations were significantly improved in the control group (*p* < 0.05). Time spent with dyskinesias was significantly worsened only in the IST group (*p* < 0.05).

### 3.3 PDQ-39

There were no significant differences in each score at weeks 0 and 36 between the IST group and control group [5]. Additionally, the total score demonstrated no significant improvement in each group [5] (Table 2, Supplementary Table S5).

Table 2 and Supplementary Table S5 show the change in the PDQ-39 subitems scores from weeks 0 to 36. Significant improvements in scores related to doing up buttons or shoelaces, writing, feeling angry or bitter, and unexpectedly falling asleep during the day were observed only in the IST group (*p* < 0.05). No significant improvement was observed in the control group.

## 4 Discussion

In this study, no significant differences in any of the scores were observed between the IST and the control groups. However, many items showed significant changes between weeks 0 and 36 in the IST group compared with the control group. Over the 36-week period, the IST group demonstrated significant improvements in MDS-UPDRS subitems including daytime sleepiness, fatigue, doing hobbies and other activities, facial expression, rigidity (right upper limb), finger tapping (right), toe tapping (right and left), time spent in the off state, and complexity of motor fluctuations, PDQ-39 scores related to doing up buttons or tying shoelaces, writing, feeling angry or bitter, and unexpectedly falling asleep during the day. By contrast, the control group showed no improvement in any PDQ-39 items, with significant improvements observed only in rigidity (left lower extremity), time spent in the off state, and functional impact of fluctuations between weeks 0 and 36. No serious adverse drug reactions occurred in either group [5]. Whereas the control group showed only limited improvement in motor symptoms, the IST group demonstrated wide-ranging improvements in both motor and non-motor symptoms.

IST significantly improved sleep abnormalities such as daytime sleepiness or unexpectedly falling asleep during the day, as previously reported [11]. Adenosine is an inhibitory neuromodulator that plays a role in regulating the sleep-wake cycle. The A_2A_ receptor plays a major role in regulating sleep among the adenosine receptors that facilitate sleep induction [18,19]. Lazarus et al. [20] indicated that caffeine induced wakefulness by antagonizing adenosine A_2A_ receptors in the nucleus accumbens, where adenosine A_2A_ receptors are highly expressed. Adenosine A_2A_ receptors stimulate the GABAergic output neurons in the indirect pathway, leading to decreased activity in the arousal systems of the thalamus, hypothalamus, brainstem, and, consequently, the cerebral cortex [18]. This suggests that the antagonistic effect of IST on adenosine A_2A_ receptors improves sleep abnormalities by maintaining arousal activity.

IST also significantly improved the ability to engage in hobbies and other activities and reduced feelings of anger or bitterness. These improvements may be partly influenced by the previously reported effects of IST on mood disorders [10]. In patients with PD, because the ventral striatum is a primary site of pathology, psychiatric symptoms such as mood disorders and anxiety can develop [21]. Therefore, the antagonistic action of IST may alleviate these mood disorders in patients with PD by modulating the function of adenosine A_2A_ receptors in the nucleus accumbens and other structures, as suggested previously [9,22]. In addition, IST has been shown to enhance dopamine levels in the prefrontal cortex in rat models [23]. An IST-induced dopamine increase may also be related to improvements in mood disturbances, but further studies are needed to clarify the mechanism.

IST resulted in significant improvements in PDQ-39 scores related to doing up buttons or shoelaces as well as writing, suggesting recovery of skill or dexterity; however, the underlying mechanism remains unclear. Adenosine A_2A_ receptors are localized in the basal ganglia, specifically within the indirect output pathway [24]. Part of the striatum contains a loop related to skill, which projects to the association cortex (supplementary motor area) via the thalamus and then loops back to a part of the putamen [24]. Therefore, IST may modulate the output from the striatum that influences motor control, leading to the enhancement of skill or dexterity.

IST also significantly improved fatigue, as previously reported [14], although the underlying mechanism is not fully understood. It has been reported that brain adenosine receptors are involved in fatigue [25] and that the ergogenic effect of caffeine is mediated by the adenosine A_2A_ receptors in the striatum [26]. This suggests that the antagonistic effect of IST on the adenosine A_2A_ receptors may contribute to the amelioration of fatigue. Because fatigue is closely associated with QOL, IST is also expected to improve the QOL of patients with PD [23].

This study had some limitations. First, it was an open-label study and susceptible to performance bias. However, the involvement of multiple centers helped to reduce investigator bias. Second, the data were collected from a restricted population consisting solely of Japanese patients. Finally, the research period was set to 37 weeks in this study. Long-term studies are necessary to assess the prolonged effects of IST on PD symptoms.

## 5 Conclusions

IST significantly improved both motor symptoms and some non-motor symptoms related to daytime sleepiness, mood, fatigue, and several QOL items; by contrast, no such widely acting effects were observed in the control group, which did not receive IST. These findings suggest that adjunctive IST may provide more comprehensive improvement in PD symptoms than would result from increasing the levodopa dose alone.

## 7 Medical Writing, Editorial, and other assistance

Medical writing support and editorial assistance in the preparation of this article were provided by Kazuyoshi Masuda, PhD, of ASCA Corporation (www.asca−co.com), and English proofreading support was also provided by ASCA Corporation, funded by Kyowa Kirin Co., Ltd.

## 2.6 Ethics

The present study was performed in compliance with the Japan Clinical Trials Act, all related national and international guidelines for human trials, and the Declaration of Helsinki. The Juntendo University Certified Review Board (J18-006) reviewed and approved the study protocol (version 6.0, 1 November 2021) and all other study documentation (approval number CRB3180012). This trial was registered with the Japan Registry of Clinical Trials (jRCTs031180248). All patients provided written informed consent before enrollment. We confirm that we have read the journal’s position on issues involved in ethical publication and affirm that this work is consistent with those guidelines.

## CRediT authorship contribution statement

**Hiroshi Nagayama:** Writing – review & editing, Writing – original draft, Methodology, Investigation, Conceptualization. **Osamu Kano:** Writing – review & editing, Methodology, Investigation, Conceptualization. **Renpei Sengoku:** Writing – review & editing, Methodology, Investigation, Conceptualization. **Naotake Yanagisawa:** Writing – review & editing, Methodology, Investigation, Formal analysis. **Asako Yoritaka:** Writing – review & editing, Investigation. **Keisuke Suzuki:** Writing – review & editing, Investigation. **Noriko Nishikawa:** Writing – review & editing, Investigation. **Yohei Mukai:** Writing – review & editing, Investigation. **Kyoichi Nomura:** Writing – review & editing, Investigation. **Norihito Yoshida:** Writing – review & editing, Investigation. **Morinobu Seki:** Writing – review & editing, Investigation. **Miho Kawabe Matsukawa:** Writing – review & editing, Investigation. **Hiroo Terashi:** Writing – review & editing, Investigation. **Katsuo Kimura:** Writing – review & editing, Investigation. **Jun Tashiro:** Writing – review & editing, Investigation. **Shigeki Hirano:** Writing – review & editing, Investigation. **Hidetomo Murakami:** Writing – review & editing, Investigation. **Hideto Joki:** Writing – review & editing, Investigation. **Tsuyoshi Uchiyama:** Writing – review & editing, Investigation. **Hideki Shimura:** Writing – review & editing, Investigation. **Kotaro Ogaki:** Writing – review & editing, Investigation. **Jiro Fukae:** Writing – review & editing, Investigation. **Yoshio Tsuboi:** Writing – review & editing, Investigation. **Kazushi Takahashi:** Writing – review & editing, Investigation. **Toshimasa Yamamoto:** Writing – review & editing, Investigation. **Kenichi Kaida:** Writing – review & editing, Investigation. **Ryoko Ihara:** Writing – review & editing, Investigation. **Kazutomi Kanemaru:** Writing – review & editing, Investigation. **Taku Hatano:** Writing – review & editing, Methodology, Investigation, Conceptualization.

## Ethics approval

The present study was performed in compliance with the Japan Clinical Trials Act, all related national and international guidelines for human trials, and the Declaration of Helsinki. The Juntendo University Certified Review Board (J18-006) reviewed and approved the study protocol (Version 6.0, 1 November 2021) and all other study documentation (approval number CRB3180012). This trial was registered with the Japan Registry of Clinical Trials (jRCTs031180248). All patients provided written informed consent before enrollment. We confirm that we have read the journal’s position on issues involved in ethical publication and affirm that this work is consistent with those guidelines.

## Funding

This work was supported by Kyowa Kirin Co., Ltd. (Tokyo, Japan). The sponsor was not involved in the study processes, including data management, monitoring/audits, statistical analysis, and interpretation of the results.

## Declaration of competing interest

The authors declare the following financial interests/personal relationships which may be considered as potential competing interests: [Hiroshi Nagayama reports grants and consultation fees from Kyowa Kirin Co., Ltd., and affiliates to endowed department (Department of Neurofunctional Analysis, Nippon Medical School, cooperated with Jusendo General Hospital). Osamu Kano reports consultation fees from AbbVie GK, Biogen Japan Ltd., Novartis Pharmaceuticals, Eli Lilly & Co., Merck Biopharma Co., Ltd., Otsuka Pharmaceutical Co., Ltd., and Kyowa Kirin Co., Ltd.; grants from Mitsubishi Tanabe Pharma Co. and Sanofi K.K.; honoraria from Mitsubishi Tanabe Pharma Co. Renpei Sengoku reports grants and consultation fees from Kyowa Kirin Co., Ltd. Naotake Yanagisawa reports grants and consultation fees from Kyowa Kirin Co., Ltd. Asako Yoritaka reports speaker’s honoraria from Kyowa Kirin Co., Ltd.; lecture fees from Sumitomo Pharma Co., Ltd., Takeda Pharmaceutical Co., Ltd., Eisai Co., Ltd., and Kyowa Kirin Co., Ltd. Keisuke Suzuki reports grants and consultation fees from Kyowa Kirin Co., Ltd.; speaker’s honoraria from Kyowa Kirin Co., Ltd., Otsuka Pharmaceutical Co., Ltd., Sumitomo Pharma Co., Ltd., Takeda Pharmaceutical Co., Ltd., Eisai Co., Ltd., Novartis Pharma K.K., and AbbVie GK. Noriko Nishikawa reports grants from Kyowa Kirin Co., Ltd. Yohei Mukai reports lecture fees from Kyowa Kirin Co., Ltd. Kyoichi Nomura reports grants from Kyowa Kirin Co., Ltd. Norihito Yoshida reports grants from Kyowa Kirin Co., Ltd. Morinobu Seki reports grants from Kyowa Kirin Co., Ltd., Daiwa Securities Foundation and Japan Philanthropic Foundation. Miho Kawabe Matsukawa reports grants from Kyowa Kirin Co., Ltd. Hiroo Terashi reports grants, consultation fees and payment or honoraria for lectures, presentations, speaker’s bureaus, manuscript writing or educational events from Kyowa Kirin Co., Ltd. Katsuo Kimura reports grants and lecture fees from Kyowa Kirin Co., Ltd.; grants from Novartis AG; and personal fees from Medtronic Japan Co., Ltd., Boston Scientific Corporation, Ono Pharmaceutical Co., Ltd., Otsuka Pharmaceutical Co., Ltd., AbbVie GK, Eisai Co., Ltd., Takeda Pharmaceutical Co., Ltd., and Sumitomo Pharma Co., Ltd. Jun Tashiro reports grants and personal fees and non-financial support from Kyowa Kirin Co., Ltd.; grants and non-financial support from CSL Behring K.K.; personal fees and non-financial support from Takeda Pharmaceutical Co., Ltd., Sumitomo Pharma Co., Ltd., FP Pharmaceutical Corporation, Ono Pharmaceutical Co., Ltd., Eisai Co., Ltd., and AbbVie GK. Shigeki Hirano reports grants from Kyowa Kirin Co., Ltd., Eli Lilly Japan K.K.; grants and lecture fees from Nihon Medi-Physics Co., Ltd.; and lecture fees from Daiichi Sankyo Co., Ltd. Hidetomo Murakami reports grants from Kyowa Kirin Co., Ltd. Hideto Joki reports grants from Kyowa Kirin Co., Ltd. Tsuyoshi Uchiyama reports grants from Kyowa Kirin Co., Ltd. Hideki Shimura reports grants and lecture fees from Kyowa Kirin Co., Ltd. Kotaro Ogaki reports grants from Kyowa Kirin Co., Ltd., JPS KAKENHI (under grants number 19 K17047); speaker’s honoraria from Sumitomo Pharma Co., Ltd., Takeda Pharmaceutical Co., Ltd., Kyowa Kirin Co., Ltd., Otsuka Pharmaceutical Co., Ltd., Ono Pharmaceutical Co., Ltd., FP Pharmaceutical Corporation, Mochida Pharmaceutical Co., Ltd., Daiichi Sankyo Co., Ltd., Eli Lilly Japan K.K., Kowa Company, Ltd., Tsumura & Co., and Eisai Co., Ltd. Jiro Fukae reports grants from Kyowa Kirin Co., Ltd. Yoshio Tsuboi reports personal fees from Eisai Co., Ltd., Takeda Pharmaceutical Co., Ltd., Novartis Pharma K.K., Sumitomo Pharma Co., Ltd., AbbVie GK, Otsuka Pharmaceutical Co., Ltd., and Kyowa Kirin Co., Ltd.; and were supported by a royalties or licenses from Nipro Corporation. Kazushi Takahashi reports grants from Kyowa Kirin Co., Ltd.; payment or honoraria for lectures, presentations, speaker’s bureaus, manuscript writing or educational events from AbbVie G.K, Ono Pharmaceutical Co., Ltd., Takeda Pharmaceutical Co., Ltd., Otsuka Pharmaceutical Co., Ltd., and Eisai Co., Ltd. Toshimasa Yamamoto reports grants from Kyowa Kirin Co., Ltd. Kenichi Kaida reports no relevant disclosures. Ryoko Ihara reports consulting fees and payment or honoraria for lectures, presentations, speaker’s bureaus, manuscript writing or educational events from Eisai Co., Ltd., and Eli Lilly K.K. Kazutomi Kanemaru reports no relevant disclosures. Taku Hatano reports grants from Kyowa Kirin Co., Ltd., Uehara Memorial Foundation, JSPS KAKENHI (under grant number 24 K02373), Japan Agency for Medical Research and Development (grant number 24dk0207072, 24wm0425015, 24bm1423015, 24dk0207055, and 24wm0425019); speaker’s honoraria from Sumitomo Pharma Co., Ltd., Takeda Pharmaceutical Co., Ltd., AbbVie Inc., Otsuka Pharmaceutical Co., Ltd., Kyowa Kirin Co., Ltd., Merck & Co., Inc., FP Pharmaceutical Corporation, and EA Pharma Co., Ltd].

## Data Availability

Hiroshi Nagayama takes responsibility for the integrity of the data and the accuracy of the data analysis. The datasets generated and/or analyzed during the current study are not publicly available because permission for their secondary use, including data sharing, has not been obtained from the participants in this study. The study protocol has been published previously [17], and the statistical analysis plan has been uploaded as a supplementary file (Data S).
