# Supplementary material for: Impact of istradefylline on motor and non-motor symptoms in patients with Parkinson’s disease: Subanalysis of the ISTRA ADJUST PD
Source: Clin Park Relat Disord. 2025 Apr 17;12:100327. doi: 10.1016/j.prdoa.2025.100327 (PMC12049841; doi:10.1016/j.prdoa.2025.100327)
Supplement: Supplementary Data 1 [file mmc1.pdf]

SUPPLEMENTARY MATERIAL

Impact of istradefylline on motor and non-motor symptoms in patients with Parkinson's disease: Subanalysis of the ISTRA ADJUST PD

Hiroshi Nagayama[a,*], Osamu Kano[b], Renpei Sengoku[c], Naotake Yanagisawa[d], Asako Yoritaka[e], Keisuke Suzuki[f], Noriko Nishikawa[g], Yohei Mukai[h], Kyoichi Nomura[i], Norihito Yoshida[j], Morinobu Seki[k], Miho Kawabe Matsukawa[l,1], Hiroo Terashi[m], Katsuo Kimura[n], Jun Tashiro[o,2], Shigeki Hirano[p], Hidetomo Murakami[q,3], Hideto Joki[r], Tsuyoshi Uchiyama[s], Hideki Shimura[e], Kotaro Ogaki[t], Jiro Fukae[u,4], Yoshio Tsuboi[v,5], Kazushi Takahashi[w], Toshimasa Yamamoto[x], Kenichi Kaida[j], Ryoko Ihara[l], Kazutomi Kanemaru[l], Taku Hatano[g]

[a]Department of Neurofunctional Science, Nippon Medical School, Tokyo, Japan

[b]Department of Neurology, Toho University Faculty of Medicine, Tokyo, Japan

[c]Department of Neurology, Daisan Hospital, The Jikei University School of Medicine, Tokyo, Japan

[d]Medical Technology Innovation Center, Juntendo University, Tokyo, Japan

[e]Department of Neurology, Juntendo University Koshigaya Hospital, Saitama, Japan

[f]Department of Neurology, Dokkyo Medical University Hospital, Tochigi, Japan

[g]Department of Neurology, Faculty of Medicine, Juntendo University, Tokyo, Japan

[h]Department of Neurology, National Center of Neurology and Psychiatry, Tokyo, Japan

[i]Department of Neurology, Higashimatsuyama Municipal Hospital, Saitama, Japan

[j]Department of Neurology, Saitama Medical Center, Saitama Medical University, Saitama, Japan

[k]Department of Neurology, Keio University School of Medicine, Tokyo, Japan

[l]Department of Neurology, Tokyo Metropolitan Institute for Geriatrics and Gerontology, Tokyo, Japan

[m]Department of Neurology, Tokyo Medical University, Tokyo, Japan

[n]Department of Neurology, Yokohama City University Medical Center, Yokohama, Japan

[o]Sapporo Parkinson MS Neurological Clinic, Sapporo, Japan

[p]Department of Neurology, Graduate School of Medicine, Chiba University, Chiba, Japan

[q]Department of Neurology, The Jikei University School of Medicine, Tokyo, Japan

[r]Department of Neurology, National Hospital Organization Yokohama Medical Center, Yokohama, Japan

[s]Department of Neurology, Seirei Hamamatsu General Hospital, Hamamatsu, Japan

[t]Department of Neurology, Juntendo University Urayasu Hospital, Chiba, Japan

[u]Department of Neurology, Juntendo University Nerima Hospital, Tokyo, Japan

[v]Department of Neurology, Fukuoka University, Fukuoka, Japan

[w]Department of Neurology, Tokyo Metropolitan Neurological Hospital, Tokyo, Japan

[x]Department of Neurology, Saitama Medical University, Saitama, Japan

***Corresponding author:** Hiroshi Nagayama

Department of Neurofunctional Science, Nippon Medical School

1-1-5 Sendagi, Bunkyo-ku, Tokyo 113-8602, Japan

Email: nagayama@nms.ac.jp

Present addresses:

[1]The Healthy Counselor’s Office, The University of Tokyo Hospital, Tokyo, Japan

[2]Department of Neurology, Hokkaido Neurosurgical Memorial Hospital, Hokkaido, Japan

[3]Department of Neurology, Showa University School of Medicine, Tokyo, Japan

[4]Rehabilitation Tsubasanoie Hospital, Tochigi, Japan

[5]Tsutsumi Clinic Fukuoka Parkinson's Disease Center, Fukuoka, Japan / Department of Neurology, Long-term Observation Research, Juntendo University, Tokyo, Japan

Supplementary Table 1. Change in MDS-UPDRS Part I scores from weeks 0 to 36

Endpoint	Change from weeks 0 to 36 (median [Q1, Q3], mean ± SD)	
	IST group	Control group
Total score	−1.0 (−3.0, 0.0)[a], −1.2 ± 3.3	0.0 (−1.0, 2.0), 0.3 ± 3.7
Cognitive impairment	0.0 (0.0, 0.0), 0.1 ± 0.5	0.0 (0.0, 0.0), 0.1 ± 0.5
Hallucinations and psychosis	0.0 (0.0, 0.0), 0.0 ± 0.4	0.0 (0.0, 0.0), −0.1 ± 0.5
Depressed mood	0.0 (0.0, 0.0), 0.0 ± 0.6	0.0 (0.0, 0.0), 0.0 ± 0.5
Anxious mood	0.0 (0.0, 0.0), 0.1 ± 0.6	0.0 (0.0, 0.0), 0.0 ± 0.4
Apathy	0.0 (0.0, 0.0), −0.1 ± 0.3	0.0 (0.0, 0.0)[a], 0.2 ± 0.5
Features of dopamine dysregulation syndrome	0.0 (0.0, 0.0), 0.0 ± 0.3	0.0 (0.0, 0.0), −0.1 ± 0.4
Sleep problems	0.0 (−1.0, 1.0), 0.0 ± 1.0	0.0 (−1.0, 0.5), 0.0 ± 0.9
Daytime sleepiness	0.0 (−1.0, 0.0)[a], −0.4 ± 0.9	0.0 (0.0, 0.0), 0.1 ± 0.9
Pain and other sensations	0.0 (−1.0, 0.0), −0.2 ± 0.9	0.0 (−1.0, 1.0), 0.0 ± 1.1
Urinary problems	0.0 (−1.0, 0.0), −0.3 ± 0.9	0.0 (0.0, 0.0), 0.1 ± 0.7
Constipation problems	0.0 (−1.0, 0.0), −0.2 ± 1.0	0.0 (−1.0, 0.0), −0.1 ± 0.8
Lightheadedness on standing	0.0 (−1.0, 0.0), −0.1 ± 1.0	0.0 (0.0, 1.0), 0.1 ± 0.9

Fatigue	0.0 (−1.0, 0.0)[a], −0.2 ± 0.7	0.0 (0.0, 0.5), 0.1 ± 0.8

[a] $p < 0.05$, Wilcoxon signed-rank test (vs. baseline [week 0]).

IST, istradefylline; MDS-UPDRS, Movement Disorder Society Unified Parkinson's Disease Rating Scale; Q, quartile; SD, standard deviation

Supplementary Table 2. Change in MDS-UPDRS Part II scores from weeks 0 to 36

Endpoint	Change from weeks 0 to 36 (median [Q1, Q3], mean ± SD)	
	IST group	Control group
Total score	0.0 (−3.0, 2.0), −0.8 ± 4.7	0.0 (−3.0, 2.0), −0.2 ± 3.6
Speech	0.0 (0.0, 0.0), 0.1 ± 0.7	0.0 (0.0, 0.0), 0.0 ± 0.6
Saliva and drooling	0.0 (−1.0, 0.0), −0.2 ± 1.0	0.0 (−1.0, 0.0), −0.2 ± 1.1
Chewing and swallowing	0.0 (0.0, 0.0), −0.1 ± 0.7	0.0 (0.0, 0.0), 0.0 ± 0.8
Eating tasks	0.0 (−1.0, 0.0), 0.0 ± 0.8	0.0 (0.0, 0.0), 0.0 ± 0.6
Dressing	0.0 (0.0, 0.0), 0.1 ± 0.7	0.0 (0.0, 0.0), 0.0 ± 0.5
Hygiene	0.0 (0.0, 0.0), 0.0 ± 0.6	0.0 (0.0, 0.0), 0.0 ± 0.5
Handwriting	0.0 (0.0, 0.0), 0.0 ± 0.7	0.0 (0.0, 0.0), 0.0 ± 0.7
Engaging in hobbies and other activities	0.0 (−1.0, 0.0)[a], −0.3 ± 1.0	0.0 (0.0, 0.0), −0.1 ± 0.8
Turning in bed	0.0 (0.0, 0.0), −0.1 ± 0.5	0.0 (0.0, 0.0), 0.1 ± 0.5
Tremors	0.0 (0.0, 0.0), 0.1 ± 0.6	0.0 (0.0, 0.0), −0.1 ± 0.7
Getting out of bed, a car, or a deep chair	0.0 (0.0, 0.0), −0.1 ± 0.9	0.0 (0.0, 0.0), −0.1 ± 0.7

Walking and balance	0.0 (0.0, 0.0),	−0.1 ± 0.8	0.0 (0.0, 0.0),	0.1 ± 0.8
Freezing	0.0 (0.0, 0.0),	0.0 ± 0.9	0.0 (0.0, 1.0),	0.1 ± 0.7

[a] $p < 0.05$, Wilcoxon signed-rank test (vs. baseline [week 0]).

IST, istradefylline; MDS-UPDRS, Movement Disorder Society Unified Parkinson's Disease Rating Scale; Q, quartile; SD, standard deviation

Supplementary Table 3. Change in MDS-UPDRS Part III scores from weeks 0 to 36

Endpoint	Change from weeks 0 to 36 (median [Q1, Q3], mean ± SD)	
	IST group	Control group
Total score	−4.0 (−8.0, −1.0)[a], −3.7 ± 7.4	−1.5 (−5.5, 2.0)[a], −2.0 ± 6.2
Speech	0.0 (0.0, 0.0), 0.0 ± 0.5	0.0 (0.0, 0.0), 0.1 ± 0.4
Facial expression	0.0 (−1.0, 0.0)[a], −0.2 ± 0.6	0.0 (0.0, 0.0), −0.1 ± 0.5
Rigidity (neck)	0.0 (0.0, 0.0), −0.1 ± 0.6	0.0 (0.0, 0.0), −0.2 ± 0.6
Rigidity (right upper limb)	0.0 (−1.0, 0.0)[a], −0.3 ± 0.8	0.0 (0.0, 0.0), −0.2 ± 0.7
Rigidity (left upper limb)	0.0 (0.0, 0.0), −0.1 ± 0.6	0.0 (0.0, 0.0), 0.0 ± 0.8
Rigidity (right lower leg)	0.0 (0.0, 0.0), −0.1 ± 0.8	0.0 (−1.0, 0.0), −0.2 ± 0.7
Rigidity (left lower leg)	0.0 (−1.0, 0.0), −0.1 ± 0.8	0.0 (−1.0, 0.0)[a], −0.2 ± 0.6
Finger tapping (right)	0.0 (−1.0, 0.0)[a], −0.2 ± 0.6	0.0 (0.0, 0.0), −0.1 ± 0.6
Finger tapping (left)	0.0 (0.0, 0.0), −0.1 ± 0.7	0.0 (0.0, 0.0), 0.0 ± 0.7
Hand movements (right)	0.0 (0.0, 0.0), −0.1 ± 0.4	0.0 (0.0, 0.0), −0.1 ± 0.5
Hand movements (left)	0.0 (0.0, 0.0), −0.1 ± 0.5	0.0 (0.0, 0.0), −0.1 ± 0.5
Pronation-supination movements of hands (right)	0.0 (−1.0, 0.0), −0.3 ± 0.9	0.0 (0.0, 0.0), 0.0 ± 0.7

Pronation-supination movements of hands (left)	0.0 (−1.0, 0.0), −0.2 ± 1.0	0.0 (0.0, 0.0), 0.0 ± 0.6
Toe tapping (right)	0.0 (−1.0, 0.0)[a], −0.3 ± 0.6	0.0 (−0.5, 0.0), 0.0 ± 0.8
Toe tapping (left)	0.0 (−1.0, 0.0)[a], −0.3 ± 0.8	0.0 (0.0, 0.0), −0.1 ± 0.5
Leg agility (right)	0.0 (0.0, 0.0), 0.0 ± 0.7	0.0 (−0.5, 0.0), −0.1 ± 0.7
Leg agility (left)	0.0 (−1.0, 0.0), 0.0 ± 0.7	0.0 (0.0, 0.0), 0.0 ± 0.7
Arising from chair	0.0 (0.0, 0.0), −0.2 ± 0.8	0.0 (0.0, 0.0), 0.0 ± 0.4
Gait	0.0 (0.0, 0.0), 0.0 ± 0.8	0.0 (0.0, 0.0), −0.1 ± 0.5
Freezing of gait	0.0 (0.0, 0.0), 0.0 ± 0.9	0.0 (0.0, 0.0), 0.0 ± 0.4
Postural stability	0.0 (−1.0, 0.0), −0.3 ± 1.0	0.0 (0.0, 0.0), −0.2 ± 0.9
Posture	0.0 (0.0, 0.0), 0.0 ± 0.8	0.0 (0.0, 0.0), 0.0 ± 0.6
Global spontaneity of movement (body bradykinesia)	0.0 (−1.0, 0.0), −0.2 ± 0.8	0.0 (0.0, 0.0), −0.1 ± 0.5
Postural tremor of the hands (right)	0.0 (0.0, 0.0), −0.1 ± 0.5	0.0 (0.0, 0.0), 0.0 ± 0.4
Postural tremor of the hands (left)	0.0 (0.0, 0.0), −0.1 ± 0.5	0.0 (0.0, 0.0), 0.0 ± 0.3
Kinetic tremor of the hands (right)	0.0 (0.0, 0.0), 0.0 ± 0.6	0.0 (0.0, 0.0), 0.0 ± 0.4
Kinetic tremor of the hands (left)	0.0 (0.0, 0.0), −0.1 ± 0.6	0.0 (0.0, 0.0), 0.0 ± 0.5

Rest tremor amplitude (right upper limb)	0.0 (0.0, 0.0),	−0.1 ± 0.5	0.0 (0.0, 0.0),	−0.1 ± 0.6
Rest tremor amplitude (left upper limb)	0.0 (0.0, 0.0),	0.0 ± 0.3	0.0 (0.0, 0.0),	0.0 ± 0.3
Rest tremor amplitude (right lower leg)	0.0 (0.0, 0.0),	0.0 ± 0.2	0.0 (0.0, 0.0),	0.0 ± 0.4
Rest tremor amplitude (left lower leg)	0.0 (0.0, 0.0),	0.0 ± 0.2	0.0 (0.0, 0.0),	0.0 ± 0.2
Rest tremor amplitude (lips/jaw)	0.0 (0.0, 0.0),	0.0 ± 0.1	0.0 (0.0, 0.0),	0.0 ± 0.1
Constancy of rest tremor	0.0 (0.0, 0.0),	−0.1 ± 0.9	0.0 (0.0, 0.0),	−0.1 ± 0.9

[a] $p < 0.05$, Wilcoxon signed-rank test (vs. baseline [week 0]).

IST, istradefylline; MDS-UPDRS, Movement Disorder Society Unified Parkinson’s Disease Rating Scale; Q, quartile; SD, standard deviation

Supplementary Table 4. Change in MDS-UPDRS Part IV scores from weeks 0 to 36

Endpoint	Change from weeks 0 to 36 (median [Q1, Q3], mean ± SD)			
	IST group		Control group	
Total score	−1.0 (−2.0, 1.0)[a],	−0.9 ± 2.4	−1.0 (−3.0, 0.0)[a],	−1.1 ± 2.5
Time spent with dyskinesias	0.0 (0.0, 0.0)[a],	0.3 ± 0.8	0.0 (0.0, 0.0),	0.1 ± 0.7
Functional impact of dyskinesias	0.0 (0.0, 0.0),	0.0 ± 0.4	0.0 (0.0, 0.0),	0.1 ± 0.4
Time spent in the off state	−0.5 (−1.0, 0.0)[a],	−0.6 ± 1.0	0.0 (−1.0, 0.0)[a],	−0.5 ± 0.9
Functional impact of fluctuations	0.0 (−1.0, 1.0),	−0.3 ± 1.2	0.0 (−1.0, 0.0)[a],	−0.3 ± 0.9
Complexity of motor fluctuations	0.0 (−1.0, 0.0)[a],	−0.3 ± 0.9	0.0 (−1.0, 0.0),	−0.3 ± 1.1
Painful off-state dystonia	0.0 (0.0, 0.0),	0.0 ± 0.7	0.0 (0.0, 0.0),	−0.1 ± 0.8

[a]$p < 0.05$, Wilcoxon signed-rank test (vs. baseline [week 0]).

IST, istradefylline; MDS-UPDRS, Movement Disorder Society Unified Parkinson's Disease Rating Scale; Q, quartile; SD, standard deviation

Supplementary Table 5. Change in PDQ-39 scores from weeks 0 to 36

Endpoint	Change from weeks 0 to 36 (median [Q1, Q3], mean ± SD)	
	IST group	Control group
Total score	0.0 (−11.0, 6.0), −3.1 ± 17.4	0.0 (−6.0, 4.0), 1.5 ± 13.2
Due to having Parkinson’s disease, how often during the last month have you…		
Had difficulty doing the leisure activities you would like to do?	0.0 (−1.0, 0.0), −0.1 ± 1.2	0.0 (−1.0, 0.0), −0.2 ± 1.1
Had difficulty looking after your home; e.g., DIY, housework, cooking?	0.0 (−1.0, 0.0), 0.0 ± 1.1	0.0 (0.0, 0.0), 0.0 ± 0.9
Had difficulty carrying bags of shopping?	0.0 (0.0, 0.0), −0.2 ± 1.1	0.0 (0.0, 0.5), 0.0 ± 1.3
Had problems walking half a mile?	0.0 (−1.0, 0.0), −0.1 ± 1.0	0.0 (−0.5, 0.0), 0.1 ± 1.3
Had problems walking 100 yards?	0.0 (0.0, 0.0), 0.1 ± 1.0	0.0 (0.0, 0.0), 0.1 ± 1.0
Had problems getting around the house as easily as you would like?	0.0 (0.0, 0.0), −0.2 ± 1.0	0.0 (0.0, 0.0), 0.0 ± 0.9
Had difficulty getting around in public?	0.0 (−1.0, 0.0), −0.3 ± 1.2	0.0 (−0.5, 0.0), −0.1 ± 1.1

Needed someone else to accompany you when you went out?	0.0 (0.0, 0.0),	−0.2 ± 0.9	0.0 (0.0, 0.0),	0.0 ± 0.8
Felt frightened or worried about falling over in public?	0.0 (0.0, 0.0),	0.0 ± 1.0	0.0 (0.0, 0.0),	0.1 ± 0.8
Been confined to the house more than you would like?	0.0 (0.0, 0.0),	0.1 ± 1.2	0.0 (0.0, 0.0),	0.0 ± 1.1
Had difficulty washing yourself?	0.0 (0.0, 0.0),	−0.1 ± 0.7	0.0 (0.0, 0.0),	0.1 ± 0.8
Had difficulty dressing yourself?	0.0 (−1.0, 0.0),	−0.2 ± 1.0	0.0 (0.0, 0.0),	0.1 ± 0.9
Had problems doing up your buttons or shoelaces?	0.0 (−1.0, 0.0)[a],	−0.3 ± 0.8	0.0 (0.0, 0.0),	0.0 ± 0.7
Had problems writing clearly?	0.0 (−1.0, 0.0)[a],	−0.4 ± 0.9	0.0 (0.0, 0.0),	0.1 ± 0.8
Had difficulty cutting food into bite-sized pieces with chopsticks or a knife?	0.0 (−1.0, 0.0),	−0.1 ± 0.8	0.0 (0.0, 0.0),	0.0 ± 1.1
Had difficulty holding a drink without spilling it?	0.0 (0.0, 0.0),	0.0 ± 1.0	0.0 (0.0, 0.0),	0.1 ± 0.7
Felt depressed?	0.0 (0.0, 0.0),	−0.1 ± 0.8	0.0 (−1.0, 0.0),	−0.1 ± 0.9
Felt isolated and lonely?	0.0 (0.0, 0.0),	0.1 ± 0.9	0.0 (0.0, 0.0),	0.1 ± 0.8
Felt weepy or tearful?	0.0 (−1.0, 0.0),	−0.1 ± 0.9	0.0 (−0.5, 1.0),	0.0 ± 1.0
Felt angry or bitter?	0.0 (0.0, 0.0)[a],	−0.2 ± 0.6	0.0 (0.0, 0.0),	0.1 ± 0.6
Felt anxious?	0.0 (0.0, 0.0),	−0.1 ± 1.0	0.0 (0.0, 0.0),	0.0 ± 0.8

Felt worried about your future?	0.0 (0.0, 0.0), 0.0 ± 1.1	0.0 (−0.5, 0.0), −0.1 ± 0.9
Felt you had to conceal your Parkinson's disease from people?	0.0 (0.0, 0.0), 0.0 ± 0.8	0.0 (0.0, 0.0), −0.1 ± 0.8
Avoided situations that involve eating or drinking in public?	0.0 (0.0, 0.0), 0.2 ± 1.2	0.0 (0.0, 0.0), 0.0 ± 1.1
Felt embarrassed in public because of having Parkinson's disease?	0.0 (0.0, 0.0), 0.1 ± 0.6	0.0 (0.0, 0.0), 0.0 ± 0.8
Felt worried by other people's reaction to you?	0.0 (0.0, 0.0), −0.1 ± 0.7	0.0 (−1.0, 0.0), −0.1 ± 0.9
Had problems with your close personal relationships?	0.0 (0.0, 0.0), 0.0 ± 0.3	0.0 (0.0, 0.0), 0.0 ± 0.6
Lacked support in the ways you need from your spouse or partner? (If you do not have a spouse or partner, the score is set to 0.)	0.0 (0.0, 0.0), −0.1 ± 0.7	0.0 (0.0, 0.0), 0.1 ± 0.8
Lacked support in the ways you need from your family or close friends?	0.0 (0.0, 0.0), −0.1 ± 0.5	0.0 (0.0, 0.0), 0.1 ± 0.6
Unexpectedly fallen asleep during the day?	0.0 (−1.0, 0.0)[a], −0.3 ± 0.9	0.0 (−1.0, 0.0), −0.1 ± 0.9
Had problems with your concentration; e.g., when reading	0.0 (0.0, 1.0), 0.0 ± 0.8	0.0 (0.0, 0.5), 0.2 ± 0.9

or watching television?				
Felt your memory was bad?	0.0 (0.0, 0.0),	0.0 ± 0.9	0.0 (0.0, 1.0),	0.1 ± 0.9
Had distressing dreams or hallucinations?	0.0 (0.0, 1.0),	0.2 ± 0.8	0.0 (0.0, 0.5),	0.1 ± 0.9
Had difficulty with your speech?	0.0 (0.0, 0.0),	−0.2 ± 0.6	0.0 (0.0, 0.0),	0.2 ± 0.6
Felt unable to communicate with people properly?	0.0 (0.0, 0.0),	0.0 ± 0.7	0.0 (0.0, 0.0),	0.1 ± 0.6
Felt ignored by people?	0.0 (0.0, 0.0),	0.0 ± 0.3	0.0 (0.0, 0.0),	0.1 ± 0.5
Had painful muscle cramps or spasms?	0.0 (−1.0, 1.0),	0.0 ± 1.1	0.0 (−1.0, 0.5),	0.0 ± 0.9
Had aches and pains in your joints or body?	0.0 (−1.0, 0.0),	0.0 ± 1.3	0.0 (0.0, 1.0),	0.1 ± 1.1
Felt unpleasantly hot or cold?	0.0 (−1.0, 0.0),	−0.2 ± 1.0	0.0 (0.0, 1.0),	0.1 ± 0.9

[a] $p < 0.05$, Wilcoxon signed-rank test (vs. baseline [week 0]).

IST, istradefylline; PDQ, Parkinson's Disease Questionnaire; Q, quartile; SD, standard deviation

Supplementary Data S

Study on the effect of istradefylline on the titration of levodopa-containing medications in Parkinson's disease patients

(Intervention study)Statistical Analysis Plan

Version 2.0

	Affiliation	Name	Signature
Preparation	Mebix, Inc.	Koichi Kigawa	Date prepared: November 16, 2021<br>Signature date:<br>Signature:
Review	Juntendo University Hospital	Naotake Yanagisawa	Date reviewed: November 18, 2021<br>Signature date:<br>Signature:
Approval	Department of Neurology, Juntendo University	Taku Hatano	Date approved: November 18, 2021<br>Signature date:<br>Signature:

*This document is in effect as of the date of approval by the approver

Preparation/Revision history

Version	Date prepared/revised	Details of revision
1.0	June 1, 2021	Preparation of first version
2.0	November 18, 2021	Revised 2.0 ver.

Table of Contents

1 PURPOSE OF THIS DOCUMENT 1
2 LIST OF ABBREVIATIONS AND DEFINITIONS OF TERMS 1
3 SOFTWARE TO BE USED 1
4 OVERVIEW OF STUDY DESIGN 1
5 TARGET ANALYSIS POPULATION 4
5.1 STUDY POPULATION 4
5.2 EFFICACY ANALYSIS SET 4
5.3 SAFETY ANALYSIS SET 4
6 HANDLING OF DATA 5
6.1 RANDOMIZATION ARMS 5
6.2 PROCEDURE FOR HANDLING MISSING, EXCLUDED, AND ABNORMAL DATA 5
6.3 HANDLING OF NUMBER OF DAYS 5
6.4 DEFINITION OF DERIVED VARIABLES 5
6.5 DEFINITION OF WEARABLE DEVICE DATA 5
6.6 HANDLING OF QUALITATIVE VARIABLES 6
7 GENERAL STATISTICAL ANALYSIS CONSIDERATIONS 6
7.1 ADJUSTMENTS OF MULTIPLICITY 6
7.2 SUMMARY STATISTICS 6
7.3 SIGNIFICANCE LEVEL OF STATISTICAL TESTS 6
7.4 NUMBER OF DIGITS TO BE EXPRESSED 7
7.5 INTERIM ANALYSIS 7
8 ANALYSIS METHOD 8
8.1 PATIENT COMPOSITION 8
8.1.1 Patient Composition 8
8.1.2 Reasons for Exclusion From Analyses 8
8.1.3 Reason for Discontinuation 8
8.2 SOCIODEMOGRAPHIC AND CLINICAL CHARACTERISTICS 8
8.2.1 Patient Baseline Characteristics 8
8.2.2 Medical History 9
8.2.3 Complications 9
8.2.4 Concomitant Drugs 9
8.3 ANALYSIS OF PRIMARY ENDPOINT 9
8.3.1 Cumulative Additional Doses of Levodopa 9
8.4 ANALYSIS OF SECONDARY ENDPOINTS 10
8.4.1 Cumulative Additional Doses of Levodopa (Week 37 Completers) 10
8.4.2 Comparison of Additional Daily Doses of Levodopa 10
8.4.3 Comparison of Number of Days to First Dose Increase of Levodopa 11
8.4.3.1 Summary Statistics and Categorical Tabulation 11

8.4.3.2 Survival Analysis 11
8.4.4 Change in CGI-S Score 12
8.4.4.1 Quantitative Variables 12
8.4.4.2 Qualitative Variables 12
8.4.5 Change in CGI-I Score 12
8.4.6 Change in PGI-S Score 12
8.4.6.1 Quantitative Variables 12
8.4.6.2 Qualitative Variables 13
8.4.7 Change in PGI-I Score 13
8.4.8 Changes in Score on the Modified Hoehn and Yahr Scale 13
8.4.8.1 ON 13
8.4.8.2 OFF 14
8.4.9 MDS-UPDRS 14
8.4.9.1 Changes in Subscores 14
8.4.9.2 Changes in Total Scores 15
8.4.10 PDQ-39 15
8.4.10.1 Changes in Subscores 15
8.4.10.2 Changes in Total Scores 15
8.4.11 Correlation of Each Score 16
8.4.12Relationship Between Cumulative Additional Doses and Background Factors (Logistic Regression Analysis) 16
8.4.12.1 Univariate Analysis 16
8.4.12.2 Multivariate Analysis 17
8.4.13 Assessment of Wearable Device Data 17
8.4.13.1 Summary Statistics 17
8.5 EXPLORATORY ENDPOINTS 18
8.5.1 Correlation of Wearable Device Data with Efficacy Endpoints 18
8.5.2 Exploration of Time Worn for Wearable Device Data 19
8.6 SAFETY EVALUATION 19
8.6.1 Occurrence of Adverse Events 19
8.6.2 Listing of Adverse Events 19
9 CHANGES FROM THE PROTOCOL 20

1 Purpose of This Document

This statistical analysis plan (hereinafter, “this document”) is prepared for the purpose of describing the details of the statistical analyses described in Section 12, “Statistical Methods,” of the clinical study, “Study on the effect of istradefylline on the titration of levodopa-containing medications in Parkinson's disease patients (Intervention study)” (hereinafter, “this study”) to be conducted by Juntendo University (hereinafter, “Juntendo”) and Kyowa Kirin Co., Ltd. (hereinafter, “Kyowa Kirin”).

The output of analysis results described in this document is described in the separately prepared document, “Statistical Analysis Figure and Table Samples.”

2 List of Abbreviations and Definitions of Terms

Abbreviations and definitions of terms used in this document are provided below.

Abbreviation	Full term
**CGI-S**	Clinical Global Impression – Severity scale
**CGI-I**	Clinical Global Impression – Improvement scale
**PGI-S**	Patient Global Impression – Severity scale
**PGI-I**	Patient Global Impression – Improvement scale
**MDS-UPDRS**	Movement Disorder Society Unified Parkinson's Disease Rating Scale
**PDQ-39**	Parkinson's Disease Questionnaire-39
**QOL**	Quality of Life
**MMSE**	Mini-Mental State Examination
**MedDRA/J**	Medical Dictionary for Regulatory Activities; Japanese version
**SOC**	System Organ Class
**PT**	Preferred Term

3 Software to be Used

The software and the version used in the statistical analyses to be performed in this study are provided below.

Software	Version
OS	Windows 10
SAS	9.4
Microsoft Office	Office 2016

4 Overview of Study Design

1) Objectives

The objectives of this study are to compare the cumulative additional doses of levodopa with and without istradefylline and to investigate the effect of istradefylline on additional doses of levodopa-containing medications (hereinafter levodopa) in patients with Parkinson’s disease taking at least 300 mg/day and no more than 400 mg/day of levodopa and are experiencing the wearing-off phenomenon. Furthermore, the efficacy endpoints (including assessments using wearable device) and safety endpoints shown below will be used to evaluate the efficacy and safety of istradefylline.

2) Study Design

A multicenter, randomized, open-label, parallel-group controlled study

3) Target Patients

Patients with Parkinson’s disease

4) Inclusion and Exclusion Criteria

- Inclusion Criteria
  1） Patients taking levodopa three times a day or more with daily dose of 300-400 mg
  2） Patients with wearing-off phenomenon
  3） Patients aged 30-84 years at enrollment
  4） Patients diagnosed with Parkinson's disease according to the diagnostic criteria of The International Parkinson and Movement Disorder Society (MDS)
  5） Patients with modified Hoehn & Yahr scale (‘ON’) stage 3 or less
  6） Patients who gave written informed consent
  For participants who have difficulty in writing due to the disease state, the consent form may be signed by a witness after obtaining oral consent from the participant

- Exclusion Criteria
  1） Patients who have taken istradefylline before
  2） Patients who received any investigational drug within 4 months before the date of enrollment
  3） Patients with dementia or Mini-Mental State Examination (MMSE) scores of 23 or less
  4） Patients who have undergone neurosurgery for Parkinson's disease (stereotactic destruction, deep brain stimulation, gamma knife, etc.)
  5） Patients receiving or scheduled to receive treatment with levodopa/carbidopa hydrate enteral suspension at enrollment
  6） Patients with moderate or severe hepatic disorder

7） Patients who had newly started treatment with anti-Parkinson's disease drugs or whose prescription had been changed (type of drug, dosage and administration) within 4 weeks before the enrollment

8） Patients who had taken drugs that strongly inhibit CYP3A4 (itraconazole, clarithromycin, etc.) within 14 days before the enrollment

9） Patients who are breastfeeding, pregnant, or possibly pregnant

10） Patients who are judged to be ineligible by the investigator or subinvestigator

5) Adjustment Factors

i) Age: ≥60 years old and <60 years old

ii) Levodopa equivalent dose: ≥400 mg/day and <400 mg/day

iii) Presence or absence of dyskinesia

6) Endpoints

(1) Primary Endpoint

Comparison of cumulative additional doses of levodopa (area under the graph of the additional dose during the treatment period)

(2) Secondary Endpoints

<Efficacy endpoints>

Comparison of additional doses per observation day from Week 4 through Week 36

Comparison of number of days to first dose increase from Week 4 on

Change in dose of levodopa up to Week 36 (after the Week 36 dose increase decision)

CGI-S score and change in CGI-S score

CGI-I (improvement from previous assessment) score

PGI-S score and change in PGI-S score

PGI-I (improvement from previous assessment) score

Modified Hoehn and Yahr grading scale (ON/OFF) score and change in modified Hoehn and Yahr grading scale (ON/OFF) score

MDS-UPDRS Part I score and change in MDS-UPDRS Part I score

MDS-UPDRS Part II score and change in MDS-UPDRS Part II score

MDS-UPDRS Part III score and change in MDS-UPDRS Part III score

MDS-UPDRS Part IV score and change in MDS-UPDRS Part IV score

PDQ-39 score and change in PDQ-39 score

Correlation of changes in above scores

< Evaluation using information extracted from data obtained by the wearable device >

The following evaluation will be performed using information extracted from accelerometric data obtained from wearable devices.

Evaluation related to movement (extracted information: (1) Frequency of movement and (2) Intensity of movement)

Evaluation related to gait (extracted information: (1) Step count, (2) Gait pitch, and (3) Balance)

Evaluation related to sleep (extracted information: (1) Bedtime, (2) Awakening time, (3) Sleep duration, (4) Sleep efficiency, (5) Sleep onset latency, (6) Frequency and intensity of movement during sleep, and (7) time spent out of bed).

<Safety Endpoints>

Adverse events and adverse drug reactions

(3) Exploratory endpoints

Evaluation will be made on the correlation between the information extracted from the acceleration data obtained from the wearable device and the efficacy endpoints described in the primary/secondary endpoints.

5 Target Analysis Population

5.1 Study Population

All patients randomized in this study will be included.

5.2 Efficacy Analysis Set

Patients of the study population except for the following will be included in the efficacy analysis set.

- Patients who do not meet the inclusion criteria or who meet exclusion criteria
- Patients who assigned to istradefylline group but does not start administration of istradefylline
- Patients who withdraw consent before the start of the observation period (Week 0)
- Other patients considered better to be excluded by the principal investigator

5.3 Safety Analysis Set

Patients of the study population except for the following will be included in the safety analysis set.

- Patients who assigned to istradefylline group but does not start administration of istradefylline
- Patients who withdraw consent before the start of the observation period (Week 0)
- Other patients considered better to be excluded by the principal investigator

6 Handling of Data

6.1 Randomization Arms

- Istradefylline treatment arm (hereinafter referred to as the “istradefylline arm”)
- Istradefylline non-treatment arm (hereinafter referred to as the “control arm”)

6.2 Procedure for Handling Missing, Excluded, and Abnormal Data

- In the event that data to be used in analyses is missing, it will be handled as missing data and will not be imputed using a statistical method.
  Furthermore, if an abnormal data occurs, such as an outlier, all data will be included in analyses, as a rule. If data is excluded, the excluded data will be identified and a clear reason for exclusion will be provided.
- However, in the event that an investigation is required into the handling of an event that was not anticipated at the start of the study for an individual patient, the handling will be described in the Criteria for Case Handling.

6.3 Handling of Number of Days

- 1 week = 7 days
- 1 year = 365.25 days

6.4 Definition of Derived Variables

1) Period Calculation

Variable	Definition
Treatment period	Treatment completion date – Treatment start date + 1
Period excluding treatment	Date completed – Date started
Age at onset of Parkinson’s disease	Date of birth (year) – Year of onset

6.5 Definition of Wearable Device Data

1) Definition of Daytime

Variable	Definition
Daytime	08:00 to 18:00

2) Data Inclusion Method

(1) For each Visit, the number of days in which the device is worn for ≥20 hours must be ≥3 days

(2) The mean of each variable will be calculated for the data included in (1) and handled as the value for the Visit

(3) An exploratory analysis will be performed for patterns other than ≥ 20 hours for daily time worn.

6.6 Handling of Qualitative Variables

Unless otherwise stated, when a quantitative or ordinal variable is to be treated as a qualitative variable, the following definitions will be used.

Variable	Category
Age	<65 years old / ≥65 years old
Age at onset of Parkinson’s disease	<65 years old / ≥65 years old
Duration of Parkinson’s disease	<5 years / ≥5 years and <7 years / ≥7 years and <10 years / ≥10 years
Duration of wearing-off phenomenon	<1 year / ≥1 year and <3 years / ≥3 years
Modified Hoehn and Yahr scale (ON, OFF)	Less than 3 / 3 or higher
Levodopa equivalent daily dose	Less than median / Median or higher

7 General Statistical Analysis Considerations

7.1 Adjustments of Multiplicity

Since there is only one primary endpoint for this study and secondary and exploratory endpoints are exploratory in nature, there will be no adjustments for multiplicity.

7.2 Summary Statistics

The summary statistics for quantitative variables include the mean, standard deviation, median, quartiles, maximum, and minimum. Summary statistics for qualitative or ordinal variables include the frequency and percentage (%). If other statistics are calculated, they will be described separately.

7.3 Significance Level of Statistical Tests

A two-tailed significance level of 5% will be used unless otherwise stated. A two-tailed 95% confidence interval will also be used to calculate the confidence interval.

7.4 Number of Digits to be Expressed

The following will apply, as a rule. Note that the number of digits expressed represents the number of digits expressed at the time of output of analysis results, and unless otherwise stated, processing such as the rounding of values will not be performed during the process of calculations.

Variable	Number of digits
P value	Rounded off from the fifth decimal place and expressed to the fourth decimal place.<br>However, values less than 0.0001 will be expressed as <0.0001.
Number of patients, number of events	Expressed as integers
Mean, standard deviation, median, and quartiles	Rounded off at the last two significant digits for the target data and expressed to one significant digit.
Maximum, minimum	Expressed to the number of significant digits for the target data.
Percentages (%)	Rounded off from the second decimal place and expressed to the first decimal place.
Statistics	Rounded off from the fourth decimal place and expressed to the third decimal place.

7.5 Interim Analysis

An interim analysis will not be performed.

8 Analysis Method

8.1 Patient Composition

8.1.1 Patient Composition

Analysis target	Study Population
Analysis details	A flow diagram of the efficacy population and the number of patients excluded and the safety population and the number of patients excluded will be prepared.

8.1.2 Reasons for Exclusion From Analyses

Analysis target	Study Population
Analysis details	A list of patients excluded from efficacy and safety analyses will be prepared.<br>A description of the reason for exclusion for each patient will also be provided.

8.1.3 Reason for Discontinuation

Analysis target	Efficacy analysis set (Overall, Istradefylline arm, Control arm)
Analysis details	If the response is "Yes" for the presence or absence of discontinuation, the number and percentage of patients will be calculated by the reason for discontinuation.

8.2 Sociodemographic and Clinical Characteristics

8.2.1 Patient Baseline Characteristics

Analysis target	Efficacy analysis set (Overall, Istradefylline arm, Control arm)
Analysis details	Summary statistics will be calculated for the patient baseline characteristics listed in Definition.<br>Refer to Section 6.6 "Handling of Qualitative Variables" for categorization of quantitative and ordinal variables, and randomization factors (Age: ≥60 years old and <60 years old; Levodopa equivalent dose : ≥400 mg/day and <400 mg/day) will also be added.
Definition	[Variables] Age, sex, height, body weight, BMI, presence or absence of a caregiver, whether or not pregnant, whether or not hospitalized, duration of Parkinson's disease (enrollment year – year of onset), age at onset of Parkinson's disease, duration of wearing-off phenomenon (enrollment year – year of onset),

	presence or absence of family history of Parkinson's disease, presence or absence of dyskinesia, MMSE score, number of years treated with levodopa (enrollment year – year started), daily levodopa dose, presence or absence of concomitant therapies, details of concomitant therapies, levodopa equivalent daily dose, modified Hoehn and Yahr scale (ON, OFF), MDS-UPDRS Part I, II, III, and IV total score, PDQ-39 total score, CGI-S, PGI-S, and device data

8.2.2 Medical History

Analysis target	Efficacy analysis set (Overall, Istradefylline arm, Control arm)
Analysis details	If the response is "Yes" for the presence or absence of medical history, the number and percentage of patients will be calculated for the details of the medical history, and the data will be presented as a listing.

8.2.3 Complications

Analysis target	Efficacy analysis set (Overall, Istradefylline arm, Control arm)
Analysis details	If the response is "Yes" for the presence or absence of complications, the number and percentage of patients will be calculated for the details of complication, and the data will be presented as a listing.

8.2.4 Concomitant Drugs

Analysis target	Efficacy analysis set (Overall, Istradefylline arm, Control arm)
Analysis details	The number and percentage of patients using each drug will be calculated for concomitant drugs by type of drug and by drug, and the data will be presented as a listing.

8.3 Analysis of Primary Endpoint

8.3.1 Cumulative Additional Doses of Levodopa

Analysis target	Efficacy analysis set (Istradefylline arm, Control arm)
Analysis details	Summary statistics of cumulative additional doses of levodopa will be calculated by group. Comparison between the groups will also be performed using the Mann Whitney U test.
Definition	[Number of treatment days]

	Number of treatment days: Date treatment completed – Date treatment started + 1<br>However, if treatment is before informed consent is obtained, the day treatment is started will be replaced by the day informed consent is obtained.<br>For Week 37 completers who are “still ongoing after completion of the observation period,” the date treatment completed will be replaced by the date equal to the “observation start date + 259 days (37 weeks)*,” and for discontinued patients, the date treatment completed will be replaced by the date of the visit immediately after discontinuation.<br>*As the date for the visit in Week 37 is a target, the actual visit may be before or after that date, and this error may affect the cumulative additional dose. To exclude this error, for Week 37 completers who are “still ongoing after completion of the observation period,” the date is to be replaced by “observation start date + 259 days” based on the premise that levodopa are to be continued.<br>[Scope of Inclusion]<br>Istradefylline arm: Sum the doses of levodopa that was increased on and after Week 4.<br>Control arm: Sum the doses of all levodopa that was increased on and after Week 0.<br>[Calculation of cumulative additional dose]<br>Number of treatment days × Daily dose

8.4 Analysis of Secondary Endpoints

8.4.1 Cumulative Additional Doses of Levodopa (Week 37 Completers)

Analysis target	Efficacy analysis set (Istradefylline arm, Control arm)
Analysis details	Summary statistics of cumulative additional doses of levodopa will be calculated by group. Comparison between the groups will also be performed using the Mann Whitney U test.

8.4.2 Comparison of Additional Daily Doses of Levodopa

Analysis target	Efficacy analysis set

Analysis details	Summary statistics will be calculated for the additional daily dose of levodopa at the start of observations and later by groups and time point.<br>Comparison between the groups of additional doses at each time point will also be performed using the Mann Whitney U test.
Definition	[Additional doses] Dose at each time point – Dose at start of observations<br>[Time points] Weeks 4, 8, 12, 16, 20, 24, 26, 30, 32, and 36, and immediately after discontinuation

8.4.3 Comparison of Number of Days to First Dose Increase of Levodopa

8.4.3.1 Summary Statistics and Categorical Tabulation

Analysis target	Efficacy analysis set (Istradefylline arm, Control arm)
Analysis details	Summary statistics will be calculated for the number of days to the first dose increase by group.
Definition	[Number of days to the first dose increase] Date of first dose increase – Date observations started

8.4.3.2 Survival Analysis

Analysis target	Efficacy analysis set (Istradefylline arm, Control arm)
Analysis details	Taking the first dose increase on or after the day following the start of treatment to be an event, the event occurrence rate in the period from the start to the first dose increase will be estimated using the Kaplan-Meier method, and the data will be plotted.<br>The event occurrence rate will be calculated every 4 weeks, and the median time to event occurrence and the 95% confidence interval will be calculated.<br>Furthermore, the log-rank test and Cox proportional hazards model will be used for survival analyses to compare event occurrence rates between istradefylline arm and control arm.
Definition	[Number of days to the first dose increase] Date of first dose increase – Date observations started<br>[Time points]<br>Week 4 (Day 28), Week 8 (Day 56), Week 12 (Day 84), Week 16 (Day 112), Week 20 (Day 140), Week 24 (Day 168), Week 28 (Day 196), Week 32 (Day 224), and Week 36 (Day 252)

8.4.4 Change in CGI-S Score

8.4.4.1 Quantitative Variables

Analysis target	Efficacy analysis set (Istradefylline arm, Control arm)
Analysis details	The CGI-S will be treated as a quantitative variable and summary statistics will be calculated by group and time point. Comparison between the groups' scores at each time point will also be performed using the Mann Whitney U test.<br>Furthermore, summary statistics of changes from Week 0 will be calculated and a within group comparison will be performed using the Wilcoxon signed-rank test.
Definition	[Time points] Weeks 0, 4, 8, 12, 16, 20, 24, 28, 32, and 36, and immediately after discontinuation

8.4.4.2 Qualitative Variables

Analysis target	Efficacy analysis set (Istradefylline arm, Control arm)
Analysis details	The CGI-S will be treated as a qualitative variable and summary statistics will be calculated by group and time point.
Definition	[Time points] Weeks 0, 4, 8, 12, 16, 20, 24, 28, 32, and 36, and immediately after discontinuation

8.4.5 Change in CGI-I Score

Analysis target	Efficacy analysis set (Istradefylline arm, Control arm)
Analysis details	Summary statistics will be calculated for the CGI-I by group and time point. Comparison between the groups' scores at each time point will also be performed using the Mann Whitney U test.
Definition	[Time points] Weeks 4, 8, 12, 16, 20, 24, 28, 32, and 36, , and immediately after discontinuation

8.4.6 Change in PGI-S Score

8.4.6.1 Quantitative Variables

Analysis target	Efficacy analysis set (Istradefylline arm, Control arm)
Analysis details	The PGI-S will be treated as a quantitative variable and summary statistics will be calculated by group and time point. Comparison between the groups' scores at each time point will also be performed using the Mann Whitney U test.

	Furthermore, summary statistics of changes from Week 0 will be calculated and a within group comparison will be performed using the Wilcoxon signed-rank test.
Definition	[Time points] Weeks 0, 4, 8, 12, 16, 20, 24, 28, 32, and 36, and immediately after discontinuation

8.4.6.2 Qualitative Variables

Analysis target	Efficacy analysis set (Istradefylline arm, Control arm)
Analysis details	The PGI-S will be treated as a qualitative variable and summary statistics will be calculated by group and time point.
Definition	[Time points] Weeks 0, 4, 8, 12, 16, 20, 24, 28, 32, and 36, and immediately after discontinuation

8.4.7 Change in PGI-I Score

Analysis target	Efficacy analysis set (Istradefylline arm, Control arm)
Analysis details	Summary statistics will be calculated for the PGI-I by group and time point. Comparison between the groups' scores at each time point will also be performed using the Mann Whitney U test.
Definition	[Time points] Weeks 4, 8, 12, 16, 20, 24, 28, 32, and 36, , and immediately after discontinuation

8.4.8 Changes in Score on the Modified Hoehn and Yahr Scale

8.4.8.1 ON

8.4.8.1.1 Quantitative Variables

Analysis target	Efficacy analysis set (Istradefylline arm, Control arm)
Analysis details	When in the ON state, the score on the modified Hoehn and Yahr scale will be treated as a quantitative variable, and summary statistics will be calculated by group and time point. Comparison between the groups' scores at each time point will also be performed using the Mann Whitney U test.<br>Furthermore, summary statistics of changes from Week 0 will be calculated and a within group comparison will be performed using the Wilcoxon signed-rank test.
Definition	[Time points] Weeks 0, 12, 24, and 36, and immediately after discontinuation

8.4.8.1.2 Qualitative Variables

Analysis target	Efficacy analysis set (Istradefylline arm, Control arm)
Analysis details	When in the ON state, the score on the modified Hoehn and Yahr scale will be treated as a qualitative variable, and summary statistics will be calculated by group and time point.
Definition	[Time points] Weeks 0, 12, 24, and 36, and immediately after discontinuation

8.4.8.2 OFF

8.4.8.2.1 Quantitative Variables

Analysis target	Efficacy analysis set (Istradefylline arm, Control arm)
Analysis details	When in the OFF state, the score on the modified Hoehn and Yahr scale will be treated as a quantitative variable, and the same analysis as in Section 8.4.8.1.1 will be performed.
Definition	[Time points] Weeks 0, 12, 24, and 36, and immediately after discontinuation

8.4.8.2.2 Qualitative Variables

Analysis target	Efficacy analysis set (Istradefylline arm, Control arm)
Analysis details	When in the OFF state, the score on the modified Hoehn and Yahr scale will be treated as a qualitative variable, and the same analysis as in Section 8.4.8.1.2 will be performed.
Definition	[Time points] Weeks 0, 12, 24, and 36, and immediately after discontinuation

8.4.9 MDS-UPDRS

8.4.9.1 Changes in Subscores

Analysis target	Efficacy analysis set (Istradefylline arm, Control arm)
Analysis details	Each subscore of MDS-UPDRS Part I, II, III, or IV will be treated as a quantitative variable, and summary statistics will be calculated by group and time point. Comparison between the groups' scores at each time point will also be performed using the Mann Whitney U test.<br>Furthermore, summary statistics of changes from Week 0 will be calculated and a within group comparison will be performed using the Wilcoxon signed-rank test.

Definition	[Time points] Weeks 0, 12, 24, and 36, and immediately after discontinuation

8.4.9.2 Changes in Total Scores

Analysis target	Efficacy analysis set (Istradefylline arm, Control arm)
Analysis details	Summary statistics will be calculated for each total score for MDS-UPDRS Part I, II, III, or IV by group and time point.<br>Comparison between the groups' scores at each time point will also be performed using the Mann Whitney U test.<br>Furthermore, summary statistics of changes from Week 0 will be calculated and a within group comparison will be performed using the Wilcoxon signed-rank test.
Definition	[Time points] Weeks 0, 12, 24, and 36, and immediately after discontinuation

8.4.10 PDQ-39

8.4.10.1 Changes in Subscores

Analysis target	Efficacy analysis set (Istradefylline arm, Control arm)
Analysis details	Each subscore of the PDQ-39 will be treated as a quantitative variable, and summary statistics will be calculated by group and time point. Comparison between the groups' scores at each time point will be performed using the Mann Whitney U test.<br>Furthermore, summary statistics of changes from Week 0 will be calculated and a within group comparison will be performed using the Wilcoxon signed-rank test.
Definition	[Time points] Weeks 0, 12, 24, and 36, and immediately after discontinuation

8.4.10.2 Changes in Total Scores

Analysis target	Efficacy analysis set (Istradefylline arm, Control arm)
Analysis details	The number and percentage of patients will be calculated for the PDQ-39 total score by group and time point.<br>A between-arm comparison of the scores at each time point will also be performed using the Mann Whitney U test.

	Furthermore, summary statistics of changes from Week 0 will be calculated and a within group comparison will be performed using the Wilcoxon signed-rank test.
Definition	[Time points] Weeks 0, 12, 24, and 36, and immediately after discontinuation

8.4.11 Correlation of Each Score

Analysis target	Efficacy analysis set (Istradefylline arm, Control arm)
Analysis details	The correlation coefficient between the CGI-S and the PGI-S, modified Hoehn and Yahr scale (ON), modified Hoehn and Yahr scale (OFF), MDS-UPDRS (Parts I, II, III, IV) score, PDQ-39 score, and device data for each factor will be calculated, and scatter diagrams will be plotted.<br>Note that all data from Week 0 to Week 36 will be used for a single analysis for both correlation coefficients and scatter diagrams, but the scores at the same time points will be used for the first and second factors, respectively.
Definition	[Time points]<br>• CGI-S; PGI-S<br>Weeks 0, 4, 8, 12, 16, 20, 24, 28, 32, and 36, and immediately after discontinuation<br>• Modified Hoehn and Yahr scale (ON, OFF); MDS-UPDRS (Parts I, II, III, IV); PDQ-39; Device data<br>Weeks 0, 12, 24, and 36, and immediately after discontinuation

8.4.12 Relationship Between Cumulative Additional Doses and Background Factors (Logistic Regression Analysis)

8.4.12.1 Univariate Analysis

Analysis target	Efficacy analysis set<br>(Overall, Week 37 completers) × (Istradefylline arm, Control arm)
Analysis details	A univariate analysis will be performed using logistic regression analysis with the explanatory variables being the variables listed in Definition and the objective variables being at least the median or less than the median cumulative additional dose.
Definition	[Explanatory variables]<br><Quantitative variables>

	BMI, daily levodopa dose, CGI-S, PGI-S, MDS-UPDRS Parts I , II, III,IV, PDQ-39, and wearable device data (Week 0)<br><Qualitative variables><br>Age, age at onset of Parkinson's disease, sex, duration of Parkinson's disease, duration of wearing-off phenomenon, modified Hoehn and Yahr scale (ON), levodopa equivalent daily dose, and presence or absence of dyskinesia

8.4.12.2 Multivariate Analysis

Analysis target	Efficacy analysis set<br>(Overall, Week 37 completers) × (Istradefylline arm, Control arm)
Analysis details	A multivariate analysis will be performed using logistic regression analysis with the explanatory variables being the variables listed in Definition and the objective variable being at least or less than the median cumulative additional dose.<br>Although basically all explanatory variables will be used, factors will be selected from the medical viewpoint of the representative investigator in the event that calculation is not possible due to insufficient number of events, etc.
Definition	[Explanatory variables]<br><Quantitative variables><br>BMI<br><Qualitative variables><br>Age, sex, duration of Parkinson's disease, modified Hoehn and Yahr scale (ON), levodopa equivalent daily dose, and presence or absence of dyskinesia

8.4.13 Assessment of Wearable Device Data

8.4.13.1 Summary Statistics

Analysis target	Efficacy analysis set (Istradefylline arm, Control arm)
Analysis details	Summary statistics will be calculated for the wearable device data listed in Definition by group and time point.<br>Comparison between the groups' scores at each time point will also be performed using the Mann Whitney U test.

	Furthermore, summary statistics of changes from Week 0 will be calculated and a within group comparison will be performed using the Wilcoxon signed-rank test.
Definition	[Time points] Weeks 0, 12, 24, and 36, and immediately after discontinuation<br>[Wearable device data]<br>Daily step count (steps), gait pitch (steps/min), gait balance, daily frequency of movement (times/min), frequency of movement while awake (times/min), frequency of movement while asleep (times/min), daily intensity of movement (METs/min), intensity of movement while awake (METs/min), intensity of movement while asleep (METs/min), time spent daily in moderate-to-intense physical activity (≥3 METs), time spent daily in light physical activity (≥1.5 METs and <3 METs), time spent daily in low physical activity (<1.5 METs), time spent without movement during daytime (min), duration of sleep, sleep efficiency (%), time spent out of bed (min), sleep onset latency (min)<br><Data using the duration of sleep according to the diary><br>Duration of sleep (hours), sleep efficiency (%), and time spent out of bed (minutes)

8.5 Exploratory endpoints

The following analyses will be performed on exploratory endpoints.

8.5.1 Correlation of Wearable Device Data with Efficacy Endpoints

Analysis target	Efficacy analysis set (Istradefylline arm, Control arm)
Analysis details	The correlation coefficient for wearable device data listed in Definition and efficacy endpoints will be calculated, and scatter diagrams will be plotted.<br>Note that all data from Week 0 to Week 36 will be used for a single analysis for both correlation coefficients and scatter diagrams, but the scores at the same time points will be used for the first and second factors, respectively.
Definition	[Time points] Weeks 0, 12, 24, and 36, and immediately after discontinuation

	[Correlation variables]<br>Number of steps per day (steps) × MDS-UPDRS II, III<br>Frequency of movement while awake (times/minute) × MDS-UPDRS II, III<br>Time by intensity of movement per day (minutes) × MDS-UPDRS II, III,<br>Sleep efficiency (%) × MDS-UPDRS 1.7,1.8,<br>Sleep efficiency (%) (Diary) × MDS-UPDRS 1.7,1.8<br>Duration of sleep (hours) × MDS-UPDRS 1.7, 1.8<br>Duration of sleep (hours) (Diary) × MDS-UPDRS 1.7, 1.8<br>Time spent without movement during daytime (minutes) × MDS-UPDRS I, II, III

8.5.2 Exploration of Time Worn for Wearable Device Data

Analysis target	Efficacy analysis set (Istradefylline arm, Control arm)
Analysis details	An exploratory investigative analysis of the required daily time worn will be performed for the analyses described in Section 8.4.13.<br>As necessary, the analyses described in Sections 8.4.12 and 8.5.1 will also be performed by changing the required daily time worn.

8.6 Safety Evaluation

8.6.1 Occurrence of Adverse Events

Analysis target	Efficacy analysis set (Overall, Istradefylline arm, Control arm)
Analysis details	Summary statistics will be calculated for the adverse event information listed in Definition.
Definition	[Adverse event information] Presence/absence of onset, Seriousness, Seriousness category, Causal relationship to study, Drug suspected of being associated, Outcome

8.6.2 Listing of Adverse Events

Analysis target	Efficacy analysis set (Overall, Istradefylline arm, Control arm)

Analysis details	Adverse events and adverse drug reactions will be tabulated by numbers and percentages of patients and number of events according to the MedDRA/J SOC and PT.
Definition	[Adverse event information] Presence/absence of onset, Seriousness, Seriousness category, Causal relationship to study, Drug suspected of being associated, Outcome

9 Changes from the Protocol

Change from the protocol will be made to the following sections.

Section No.	Details	Reason
4 Overview of Study Design - Item 6) Endpoints	"Number of episodes of getting out of bed" changed to "Time spent out of bed"	The representative investigator judged it more appropriate for the time spent out of bed to be used based on the characteristics of the device.

End
